# Pharmacological chaperones for the oxytocin receptor increase oxytocin responsiveness in myometrial cells

**DOI:** 10.1016/j.jbc.2022.101646

**Published:** 2022-01-28

**Authors:** Manasi Malik, Yingye Fang, Monali Wakle-Prabagaran, Michelle Roh, Kevin Prifti, Antonina I. Frolova, Princess I. Imoukhuede, Sarah K. England

**Affiliations:** 1 Department of Obstetrics and Gynecology, Center for Reproductive Health Sciences, Washington University in St. Louis, St. Louis, Missouri, USA; 2 Department of Biomedical Engineering, Washington University in St. Louis, St. Louis, Missouri, USA

**Keywords:** oxytocin, oxytocin receptor, myometrium, pharmacological chaperones, pregnancy, AVPR2, arginine vasopressin receptor 2, ER, endoplasmic reticulum, FBS, fetal bovine serum, GPCR, G-protein-coupled receptor, hTERT-HM, hTERT-immortalized human myometrial, IP1, inositol monophosphate, OXTR, oxytocin receptor, PDI, protein disulfide isomerase

## Abstract

Oxytocin is a potent uterotonic agent administered to nearly all patients during childbirth in the United States. Inadequate oxytocin response can necessitate Cesarean delivery or lead to uterine atony and postpartum hemorrhage. Thus, it may be clinically useful to identify patients at risk for poor oxytocin response and develop strategies to sensitize the uterus to oxytocin. Previously, we showed that the V281M variant in the oxytocin receptor (*OXTR*) gene impairs OXTR trafficking to the cell surface, leading to a decreased oxytocin response in cells. Here, we sought to identify pharmacological chaperones that increased oxytocin response in cells expressing WT or V281M OXTR. We screened nine small-molecule agonists and antagonists of the oxytocin/vasopressin receptor family and identified two, SR49059 and L371,257, that restored both OXTR trafficking and oxytocin response in HEK293T cells transfected with V281M OXTR. In hTERT-immortalized human myometrial cells, which endogenously express WT OXTR, treatment with SR49059 and L371,257 increased the amount of OXTR on the cell surface by two- to fourfold. Furthermore, SR49059 and L371,257 increased the endogenous oxytocin response in hTERT-immortalized human myometrial cells by 35% and induced robust oxytocin responses in primary myometrial cells obtained from patients at the time of Cesarean section. If future studies demonstrate that these pharmacological chaperones or related compounds function similarly *in vivo*, we propose that they could potentially be used to enhance clinical response to oxytocin.

The nonapeptide hormone oxytocin modulates social behavior, mediates the lactation reflex, and induces and strengthens uterine contractions. Synthetic oxytocin is administered to induce or augment labor and prevent postpartum hemorrhage in a large portion of patients who give birth (1, 2). However, response to oxytocin varies widely between individuals (3). Inadequate response to oxytocin poses significant clinical challenges, including labor arrest, requirement for Cesarean section, and postpartum hemorrhage, which can increase risk for complications and lead to maternal mortality (4, 5, 6). Thus, it may be clinically advantageous to develop strategies to improve oxytocin response in laboring patients.

One strategy could be to identify pharmacological chaperones (pharmacoperones) that increase cell surface localization and function of the oxytocin receptor (OXTR), which is a G-protein-coupled receptor (GPCR). Studies on other GPCRs show that antagonists can increase cell surface levels of WT or variant receptors. For instance, long-term treatment with β adrenergic receptor antagonists (β blockers) increases receptors on the cell surface, leading to excessive β adrenergic stimulation when β blockers are withdrawn (7). Antagonist pharmacoperones appear to act by promoting anterograde transport of receptors to the cell surface: they permeate the cell membrane and bind to immature receptors either during or after translation, thus stabilizing the native state of the protein (8, 9).

Pharmacoperones have also been investigated for therapeutic use in patients with genetic variants in the arginine vasopressin receptor 2 (AVPR2), a GPCR with high similarity to OXTR (10, 11). Pathogenic variants impair AVPR2 folding and trafficking to the cell surface, resulting in nephrogenic diabetes insipidus (12). Previous investigators have shown that antagonists, agonists, and allosteric ligands are effective in rescuing AVPR2 trafficking and function: one such antagonist, SR49059, showed promise in a small clinical trial of patients with nephrogenic diabetes insipidus (13, 14). Given the similarity between AVPR2 and OXTR, compounds that act as OXTR pharmacoperones are likely to exist. However, none have been described.

Here, to identify pharmacoperones that can increase oxytocin response, we turned to the OXTR variant V281M, which is located in the sixth transmembrane domain and is most commonly found in the Swedish population (15). We previously reported that, similar to pathogenic variants in AVPR2, V281M impairs OXTR trafficking to the cell surface and significantly decreases cellular response to oxytocin (16). For the present study, we began by determining the effects of candidate pharmacoperones on V281M OXTR trafficking and function in transfected HEK293T cells. Second, we confirmed that selected compounds had similar effects on WT OXTR in HEK293T cells. Third, we examined the effect of pharmacoperones on OXTR trafficking and oxytocin response in an immortalized human myometrial cell line that endogenously expresses WT OXTR. Finally, we evaluated the ability of pharmacoperones to augment oxytocin response in primary human myometrial cells from pregnant patients.

## Results

### Small-molecule OXTR antagonists rescue trafficking and functional defects of V281M OXTR

**Figure 1:**
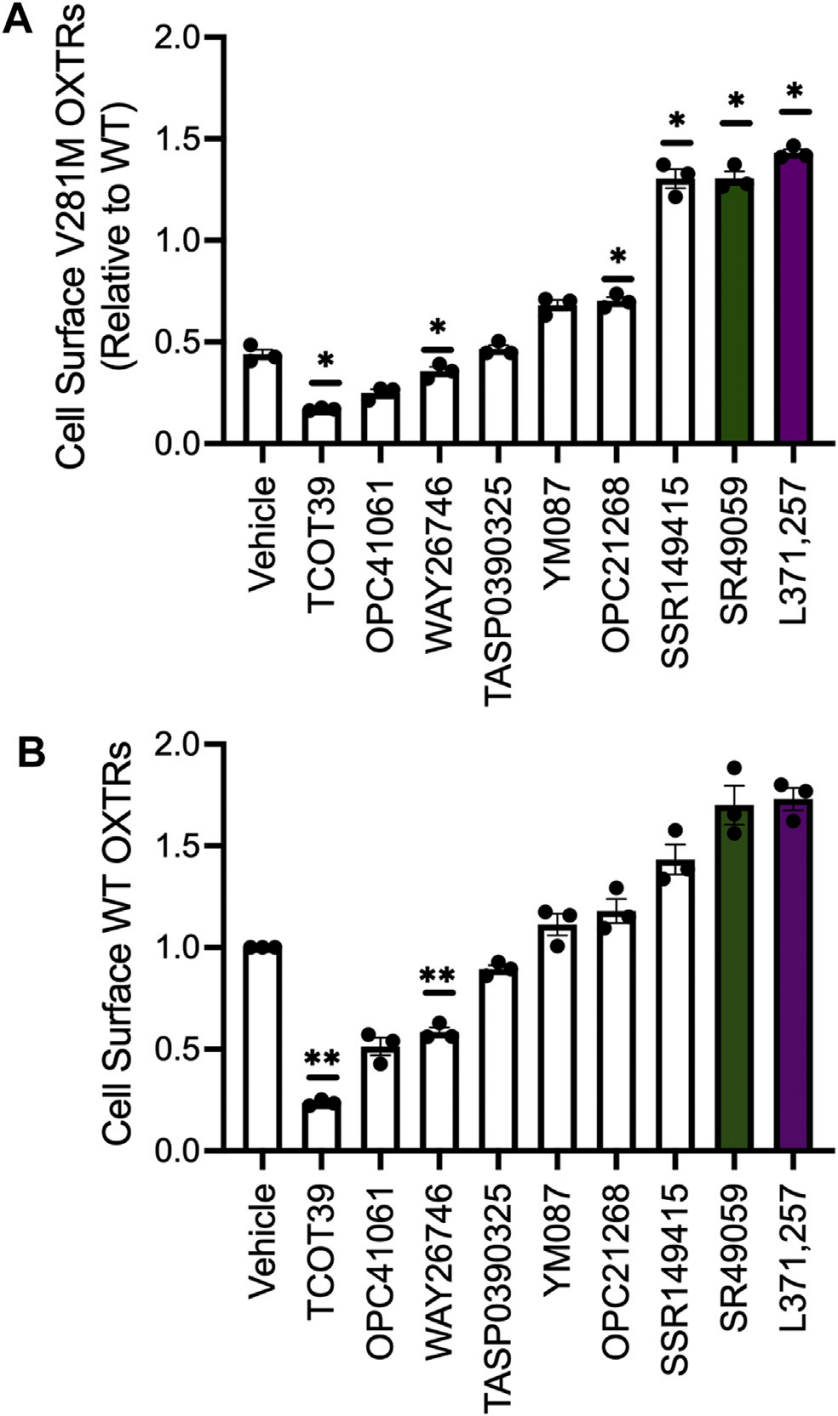
**SR49059 and L371,257 increase cell surface abundance of variant and wild-type oxytocin receptor in HEK293T cells.***A*, effects of compounds on V281M OXTR cell surface abundance in HEK293T cells plotted relative to cell surface OXTR abundance in cells transfected with WT OXTR and treated with vehicle. ∗*p* < 0.05 compared with vehicle-treated cells by one-way ANOVA with Dunnett’s multiple comparisons test. *B*, effects of compounds on WT OXTR cell surface abundance relative to that in vehicle-treated cells. ∗∗*p* < 0.0055 compared with vehicle by one-sample *t* test (α corrected for multiple comparisons by Bonferroni method). Data shown are mean and standard error from *N* = 3 independent trials. OXTR, oxytocin receptor.

To identify molecules with pharmacoperone activity, we screened nine commercially available small-molecule ligands that bind the oxytocin and/or vasopressin receptor (Table 1). We included both agonists and antagonists with varying reported affinities for OXTR. Given their high calculated lipophilicity values (logP > 2.8), all tested compounds were predicted to permeate the cell membrane (17, 18). We transiently transfected HEK293T cells with V281M OXTR tagged with hemagglutinin and GFP (HA-OXTR-GFP), treated them with 10 μM of each candidate drug for 16 h, then performed quantitative flow cytometry to measure cell surface OXTR (16, 19). Vehicle-treated cells transfected with V281M HA-OXTR-GFP had 57% fewer OXTRs on the cell surface than vehicle-treated cells transfected with WT HA-OXTR-GFP (Fig. 1*A*). The two known OXTR agonists—TCOT39 and WAY26746—decreased cell surface V281M OXTR, likely due to β-arrestin-induced internalization after OXTR activation (20, 21). TASP0390325, which has extremely low affinity for OXTR (22), had no effect on cell surface V281M OXTR. In contrast, three antagonists—SSR1494155, SR49059, and L371,257—increased cell surface V281M OXTR to 130 to 143% of WT levels (Fig. 1, *p* = 0.019, 0.012, 0.004, respectively).

**Table 1:** Candidate pharmacoperones screened for effects on OXTR cell surface expression

Drug	Primary action
TCOT39	Selective OXTR partial agonist (32)
OPC41061	AVPR2 antagonist; unknown OXTR affinity (33)
WAY26746	Selective OXTR agonist (34)
TASP0390325	Selective AVPR1A antagonist; low OXTR affinity (22)
YM087	AVPR1A/2 antagonist; medium OXTR affinity (35, 36)
OPC21268	AVPR1 antagonist; medium OXTR affinity (36)
SSR149415	AVPR1B antagonist; medium OXTR affinity (37)
SR49059	AVPR1A antagonist; medium OXTR affinity (36)
L371,257	Selective OXTR antagonist (32, 36, 38)

Numbers in parentheses are references.

We next examined the effect of these same compounds in cells transfected with WT HA-OXTR-GFP. Most had similar effects as they did on V281M OXTR (Fig. 1*B*). Cells treated with two compounds—SR49059 and L371,257—had 170% more surface WT OXTR than vehicle-treated cells (Fig. 1*B*). Therefore, we focused on these two compounds in further experiments.

**Figure 2:**
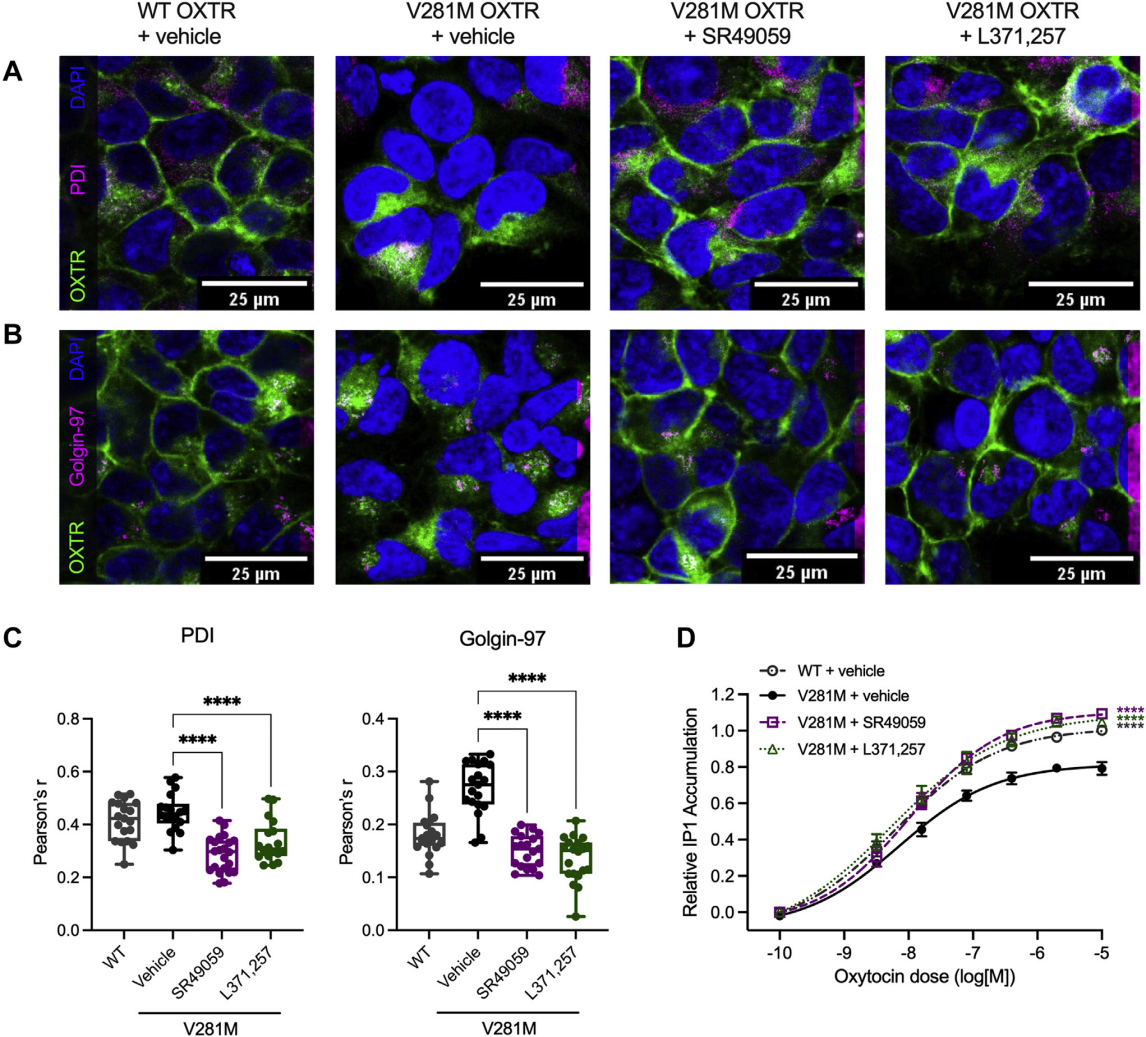
**SR49059 and L371,257 rescue trafficking and functional defects of V281M OXTR.***A*–*C*, subcellular localization of OXTR (*green*) and (*A*) ER marker PD1 (*magenta*) or (*B*) Golgi marker Golgin-97 (*magenta*) in HEK293 cells stably transfected with WT OXTR-GFP and V281M OXTR-GFP. Results shown are representative from three independent trials. *C*, quantitation of colocalization by Pearson’s r. Each point represents an individual image. ∗∗∗∗*p* < 0.0001 by one-way ANOVA with Dunnett’s multiple comparisons test. *D*, oxytocin-induced IP1 production in HEK293T cells transfected with WT or V281M OXTR and treated with vehicle or SR49059. Data are shown as mean and standard error from *N* = 5 independent trials. ∗∗∗∗*p* < 0.0001 compared with V281M + vehicle by sum-of-squares F test. OXTR, oxytocin receptor.

We next used confocal microscopy to determine the effect of SR49059 and L371,257 on subcellular localization of V281M OXTR-GFP in stably transfected HEK293 cells. In vehicle-treated cells, V281M OXTR-GFP accumulated intracellularly (Fig. 2, *A* and *B*). Costaining revealed that intracellular V281M OXTR-GFP colocalized with markers for the endoplasmic reticulum (ER) (protein disulfide isomerase, PDI) and Golgi (golgin-97). Treatment with 10 μM SR49059 or L371,257 decreased colocalization of V281M OXTR-GFP with both PDI and golgin-97 (*p* < 0.0001). We concluded that SR49059 and L371,257 allowed V281M OXTR-GFP to traffic through the ER and Golgi to reach the cell membrane, consistent with flow cytometry results.

We hypothesized that the enhanced V281M OXTR cell surface localization in SR49059-and L371,257-treated cells would lead to increased oxytocin response. To test this idea, we transiently transfected HEK293T cells with V281M OXTR, incubated them with oxytocin, and quantified inositol monophosphate (IP1), a downstream product of signaling through the Gq-phospholipase C-inositol triphosphate pathway. At baseline (no oxytocin stimulation), IP1 concentration was similar in cells transfected with WT OXTR and V281M OXTR (Fig. S1*A*). Treatment with SR49059 slightly decreased basal IP1 concentrations in V281M OXTR-transfected cells (*p* = 0.020); treatment with L371,257 had a similar, but not statistically significant, effect (*p* = 0.057, Fig. S1*A*). Stimulation with oxytocin resulted in a dose-dependent increase in IP1 concentration in all cells (Fig. 2*D*). Consistent with our previous results (16), maximal oxytocin-induced IP1 production in vehicle-treated V281M OXTR-expressing cells was 80% of that in WT OXTR-expressing cells (Fig. 2*D*). However, treatment with SR49059 and L371,257 abolished this difference (Fig. 2*D*); IP1 concentrations in oxytocin-stimulated cells were similar in V281M OXTR-expressing cells treated with SR49059 or L371,257 and WT OXTR-expressing cells treated with vehicle control (Fig. S1*B*). Taken together, these data showed that SR49059 and L371,257 restored both OXTR cell surface localization and oxytocin response in V281M OXTR-expressing cells.

### SR49059 and L371,257 increase WT OXTR cell surface localization in immortalized human myometrial cells

**Figure 3:**
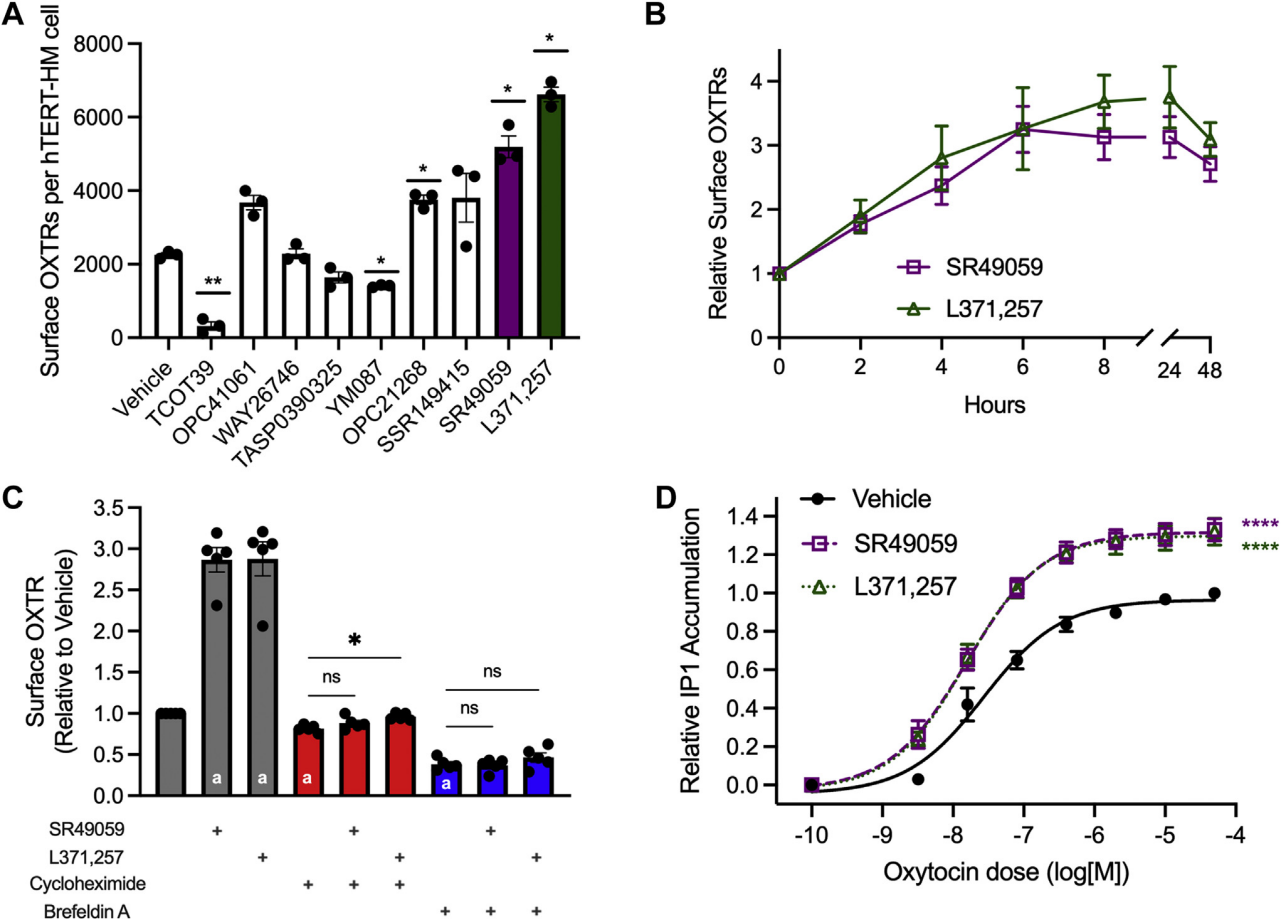
**SR49059 and L371,257 increase OXTR trafficking and oxytocin response in immortalized human myometrial cells.***A*, effect of compounds on cell surface localization of OXTR in hTERT-HM cells. Data shown are mean and standard error from *N* = 3 independent trials. ∗*p* < 0.05, ∗∗*p* < 0.01 by one-way ANOVA with Dunnett’s multiple comparisons test. *B*, change in surface OXTR on hTERT-HM cells after incubation with SR49059 or L371,257 for the indicated time points. Error bars show standard error for *N* = 5 (SR49059) and *N* = 3 (L371,257). *C*, change in surface OXTR after 12-h incubation with SR49059, L371,257, cycloheximide, and brefeldin A as indicated. Bars with “a” are statistically different from vehicle-treated cells at *p* < 0.001 (one-sample *t* test). ∗*p* < 0.05 by one-way ANOVA with Šidák’s multiple comparisons test; ns, not significant. *D*, oxytocin-induced IP1 production in hTERT-HM cells treated with SR49059 or L371,257. Data are shown as mean and standard error from *N* = 5 independent trials. ∗∗∗∗*p* < 0.0001 compared with vehicle-treated cells by sum-of-squares F test. OXTR, oxytocin receptor.

Given the robust effects of SR49059 and L371,257 in HEK293T cells, we wondered whether these compounds could enhance trafficking of WT OXTR endogenously expressed in hTERT-immortalized human myometrial (hTERT-HM) cells (23), which endogenously express WT OXTR. Because specific antibodies to OXTR are not commercially available, we used CRISPR-Cas9 to introduce an HA tag at the N-terminus of OXTR in the hTERT-HM cell line. We then performed quantitative flow cytometry to assess the effect of the nine candidate pharmacoperones on cell surface HA-OXTR (16 h treatment, 10 μM). As in HEK293T cells, the OXTR partial agonist TCOT39 decreased surface abundance of OXTRs (*p* = 0.009). However, the OXTR agonist WAY26746, which had decreased surface OXTRs in transfected HEK293T cells, had no effect in hTERT-HM cells. The AVPR1A/2 antagonist YM087, which had no effect on OXTR in HEK293T cells, significantly decreased surface OXTR in hTERT-HM cells (*p* = 0.018). The top two candidate pharmacoperones, SR49059 and L371,257, had similar effects in hTERT-HM cells as in HEK293T cells, increasing cell surface OXTR abundance by 2.3-fold and 2.9-fold, respectively (Fig. 3*A*). Surface OXTR localization increased by more than twofold after 4 h of treatment with SR49059 or L371,257, reached a plateau after 6 h of treatment, and remained high for 48 h of treatment (Fig. 3*B*).

To determine the mechanism of action of SR49059 and L371,257, we treated hTERT-HM cells for 12 h with cycloheximide (25 μg/mL), which inhibits protein translation, and brefeldin A (5 μg/mL), which blocks protein transport from the endoplasmic reticulum to the Golgi. Cycloheximide and brefeldin A decreased baseline cell surface OXTR abundance by 18% and 62%, respectively (Fig. 3*C*; *p* < 0.001, one-sample *t* test). Concurrent treatment with 10 μM SR49059 had no effect on cell surface OXTR in cells that were also treated with cycloheximide or brefeldin A. L371,257 increased surface OXTR by 20% (*p* = 0.02, one-way ANOVA with Šidák’s multiple comparisons) in cells treated with cycloheximide but had no effect on cells treated with brefeldin A (Fig. 3*C*). We concluded that both SR49059 and L371,257 primarily act by mobilizing newly synthesized OXTRs to the cell membrane, and that L371,257 may traffic a small portion of previously synthesized receptors to the cell membrane.

### SR49059 and L371,257 increase oxytocin-induced IP1 production in immortalized and primary human myometrial cells

Given the abilities of SR49059 and L371,257 to increase WT OXTR cell surface localization in hTERT-HM cells, we asked whether these pharmacoperones would also lead to increased oxytocin response in these cells. We measured oxytocin-induced IP1 production in the absence and presence of these compounds and found that SR49059 and L371,257 increased maximal IP1 production by 37% and 35%, respectively (Fig. 3*D*).

**Figure 4:**
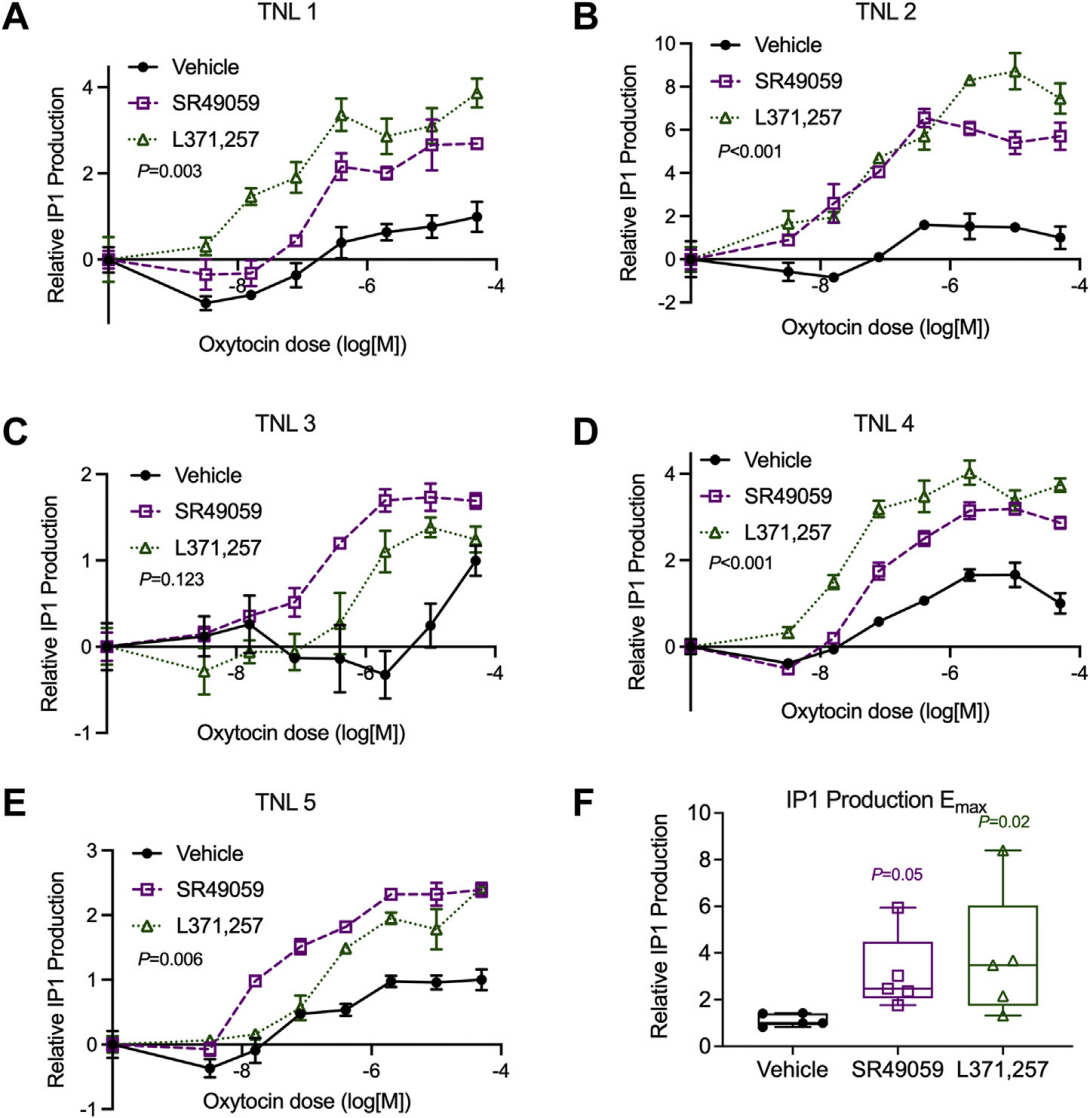
**SR49059 and L371,257 increase oxytocin-induced IP1 production in primary human myometrial cells.***A*–*E*, oxytocin-induced IP1 production in primary human myometrial samples from five term nonlaboring samples (TNL 1–5). Error bars show standard error from *N* = 4 technical replicates. *p*-values from comparison of curve E_max_ (sum-of-squares F test). *F*, E_max_ from *N* = 5 primary cell samples. *p* values from Friedman ANOVA with Dunn’s multiple comparisons test.

Finally, we asked whether SR49059 and L371,257 would affect oxytocin response in primary myometrial cells isolated from uterine tissue collected from pregnant patients at the time of term elective Cesarean section. We incubated these cells with SR49059 or L371,257 and quantified IP1 production. In four of the five primary cultures, cells treated with SR49059 and oxytocin accumulated between 1.9 and 7.0 times more IP1 than cells treated with vehicle and oxytocin. Likewise, L371,257 increased response to oxytocin by 1.9- to 5.9-fold (Fig. 4). We conclude that, although the magnitude of the effect varied widely, pharmacoperone treatment increased cellular response in primary myometrial cells.

## Discussion

Taken together, our results indicate that two oxytocin/vasopressin antagonists, SR49059 and L371,257, act as pharmacoperones for both variant and WT OXTR. First, these pharmacoperones restored OXTR trafficking and oxytocin response in HEK293T cells transfected with V281M OXTR, a loss-of-function variant. Second, pharmacoperones mobilized endogenous WT OXTR to the cell surface in a translation-dependent manner. Finally, SR49059 and L371,257 increased response to oxytocin in both hTERT-HM cells and primary human myometrial cells from five individuals.

Our report adds to several studies demonstrating the use of pharmacoperones for the closely related AVPR2 (14). Of the AVPR2 pharmacoperones that have been described (13, 24, 25, 26, 27), we included the agonists SR49059, YM087, and OPC41061 in our studies. SR49059 robustly increased cell surface OXTR in HEK293T cells transfected with V281M or WT OXTR, as well as in hTERT-HM cells. However, YM087 and OPC41061 had mixed effects. YM087 did not alter surface OXTR in HEK293T cells but decreased surface OXTR abundance in hTERT-HM cells, whereas OPC41061 increased cell surface OXTR in hTERT-HM cells but not in transfected HEK293T cells. In contrast, the OXTR antagonist L371,257, which has not previously been tested for pharmacoperone activity, had the greatest effect of all the candidate compounds on cell surface OXTR in both transfected HEK293T cells and hTERT-HM cells. It is unclear whether L371,257 also acts as a pharmacoperone for AVPR2 or other vasopressin receptors. Given the similarity between members of the oxytocin/vasopressin receptor family, agents that act on OXTR will likely also affect vasopressin receptors; however, the clinical significance of such effects is unknown.

One limitation of our study was that the lack of commercially available OXTR-specific antibodies prevented us from examining localization of endogenous, untagged OXTR. However, we found that SR49059 and L371,257 increased OXTR trafficking to the cell surface regardless of whether OXTR was tagged with an N-terminal HA tag, C-terminal GFP tag, or both. Importantly, we assessed the functional effects of SR49059 and L371,257 on oxytocin signaling in primary myometrial cells expressing endogenous, untagged OXTR.

In the long term, development of OXTR pharmacoperones may improve the safety and effectiveness of oxytocin use during childbirth. Oxytocin is administered to most patients to induce and augment labor and prevent postpartum hemorrhage. However, patients with reduced OXTR function may not benefit from oxytocin treatment. Our data show that pharmacoperone treatment can reverse the effects of a loss-of-function *OXTR* variant, potentially enabling the use of oxytocin in patients with V281M or other variants that disrupt OXTR trafficking.

In addition to their effects on V281M OXTR, SR49059 and L371,257 increased the abundance of WT OXTR on the cell surface in both transfected HEK293T and hTERT-HM cells. This suggests that pharmacoperone treatment can increase oxytocin responsiveness in all patients, regardless of their *OXTR* genotype. In support of this idea, we show that five primary myometrial samples acquired robust oxytocin responses after treatment with SR49059 and L371,257. Although we did not have sequencing data for the patients from whom we obtained these samples, it is unlikely that they harbored the V281M variant, which is most prevalent in the Swedish population (15) and found in 0.2% of non-Finnish Europeans (28). Instead, they most likely had WT OXTR.

Several questions must be addressed before OXTR pharmacoperones can be translated to the clinic. Whereas SR49059 and L371,257 increased surface OXTRs by approximately two- to fourfold, these treatments only increased OXTR signaling by 1.35-fold. This could indicate that a subset of OXTRs mobilized to the cell surface were nonfunctional, perhaps due to residual binding of SR49059 and L371,257, which are competitive antagonists. Treatment with SR49059 and L371,257 also decreased IP1 abundance in unstimulated (non-oxytocin-treated) cells, which might be the result of decreased basal (unliganded) OXTR signaling or decreased cellular health. Future experiments should investigate the effects of the antagonist activities of SR49059 and L371,257 on overall oxytocin signaling, particularly *in vivo*. If pharmacoperones that lack antagonist activity are developed and proven safe and effective in labor, they could be used to improve oxytocin sensitivity in patients predicted to have reduced oxytocin responses due to genetic or clinical factors.

## Experimental procedures

### Compounds and plasmids

Oxytocin stock solutions (Tocris) were diluted to 500 μM in water and stored at −80 °C until just before use. Stock solutions for all candidate pharmacoperones (Table 1, Tocris) were diluted to 5 mM in DMSO and stored at −80 °C until just before use. Cycloheximide (Millipore Sigma) was diluted to 10 mg/ml in DMSO and stored at −20 °C until just before use. Brefeldin A stock solution (5 mg/ml) was obtained from Biolegend. An equivalent volume of DMSO was used as a vehicle control for all experiments. Plasmids encoding WT and V281M OXTR, OXTR-GFP, and HA-OXTR-GFP were generated as previously described (16).

### Cell lines

All cell lines were maintained in Dulbecco’s Modified Eagle Medium (DMEM)/Ham’s F12 media without phenol red supplemented with 10% fetal bovine serum (FBS) and 25 μg/ml gentamicin. Cells were kept in a humidified cell culture incubator at 37 °C with 5% CO_2_. Stably transfected HEK293 cell lines expressing WT and V281M OXTR-GFP were selected and maintained with 500 μg/ml G418 (Millipore Sigma). The hTERT-HM cell line was a kind gift from Dr Jennifer Condon at Wayne State University (23).

### Primary human myometrial cell cultures

Human myometrial tissue samples from the lower uterine segment were obtained from nonlaboring patients at >37 weeks’ gestation during Cesarean section under spinal anesthesia. Human subjects research was performed in accordance with the principles stated in the Declaration of Helsinki. Participants signed consent forms approved by the Washington University in St Louis Internal Review Board (protocol no. 201108143). Tissues were cut into small pieces and incubated with 1 mg/ml collagenase IA and collagenase XI (Millipore Sigma) for 45 to 60 m at 37 °C with rotation. The collagenase-treated mixture was then passed through a 70 μm cell strainer, and collagenase was neutralized with 10% FBS. Cells were centrifuged, resuspended, and plated in DMEM/Ham’s F12 media without phenol red, supplemented with 5% Smooth Muscle Cell Growth Medium 2 (PromoCell). Cells were used at passage 0, 1, or 2.

### Quantitative flow cytometry

HEK293T cells were plated in T25 flasks (10^6^ cells/flask) and transfected the next day with 300 ng of plasmid DNA encoding HA-OXTR-GFP and 4 μl of TransIT-LT1 reagent (Mirus). Eight hours after transfection, candidate pharmacoperone or vehicle was added to each flask to a final concentration of 10 μM. Sixteen hours later, cells were detached with CellStripper (Corning) and analyzed by flow cytometry.

The Genome Engineering and iPSC Center at Washington University in St Louis used CRISPR-Cas9 to introduce an HA tag at the N-terminus of the *OXTR* gene in hTERT-HM cells. After drug incubation, cells were detached with TrypLE Express (Thermo Fisher) before flow cytometry.

Labeling and flow cytometry quantitation of cell surface HA-OXTR-GFP were performed as previously described (16, 19). An empirically determined saturating concentration of phycoerythrin-conjugated anti-HA antibody (901518, Biolegend) was used for each cell type (16 ng/μl for transfected HEK293T cells, 10 ng/μl for hTERT-HM cells).

### Immunofluorescence

HEK293T cells stably transfected with V281M OXTR-GFP were plated in poly-D-lysine-coated 8-well culture slides. After drug treatment, cells were washed once with PBS, then fixed with 4% paraformaldehyde for 20 min at room temperature. Cells were washed once with PBS, incubated in blocking and permeabilization buffer (5% FBS in PBS plus 0.1% Tween-20) for 1 h at room temperature, then incubated with anti-PDI and anti-golgin-97 antibodies (C81H6 and D82PK, respectively, 1:100, Cell Signaling Technologies) overnight at 4 °C. Cells were washed three times with PBS plus 0.1% Tween-20, then incubated with goat anti-rabbit secondary antibody (1:1000, AlexaFluor 555, Thermo Fisher) for 1 h at room temperature. Cells were washed and nuclei labeled with NucBlue fixed cell stain (Thermo Fisher) before imaging by confocal microscopy (Leica DM4000). Investigators were masked to treatment when imaging slides. Colocalization (Pearson’s r) was calculated by using the JaCoP plugin in ImageJ software (29).

### IP1 production

IP-One Gq Homogeneous Time Resolved Fluorescence kit (Cisbio) was used according to the manufacturer’s instructions to measure IP1 production. To measure IP1 production in transfected HEK293T cells, cells were transfected in T25 flasks as above. Eight hours later, cells were treated with 10 μM SR49059, 10 μM L371,257, or 0.2% DMSO (vehicle). Sixteen hours later, cells were washed extensively and detached with TrypLE Express. Cell suspensions were incubated with the indicated doses of oxytocin (60,000 cells in a total volume of 15 μl cell suspension per well) for 1 h at 37 °C. After lysis and addition of Homogeneous Time Resolved Fluorescence donor and acceptor, plates were incubated for 1 h at room temperature. Fluorescence was read on a PerkinElmer Envision plate reader. The same protocol was used to measure IP1 production in hTERT-HM cells and primary human myometrial cells, but 5000 to 7500 cells were used per well.

Nonlinear regression with least-squares fitting was used to generate dose–response curves with the following model:

$$\text{Y}=\text{Bottom}+\left(\text{Top}-\text{Bottom}\right)/\left(1+10^{\wedge }\left(\left(\text{LogEC}50\;\text{or}\;\text{IC}50-\text{X}\right)*\text{HillSlope}\right)\right)\left(\text{GraphPad}\;\text{Prism}\right)$$

In this model, Y = response, X = log(oxytocin concentration), and no constraints were placed on any values. E_max_ values were compared by performing nested extra sum-of-squares F-tests as previously described (30, 31).

## Data availability

All data are contained within the article and Supporting Information.

## Supporting information

This article contains supporting information.

## Conflict of interest

M. M., Y. F., P. I. I., and S. K. E. have filed a provisional patent application (US 63/209,054) for compositions and methods for increasing cell surface OXTR. S. K. E., M. M., P. I. I., and Y. F. have patent #US 63/209,054 pending to Washington University in St. Louis.
