# Supplementary material for: Pharmacological chaperones for the oxytocin receptor increase oxytocin responsiveness in myometrial cells
Source: J Biol Chem. 2022 Jan 28;298(3):101646. doi: 10.1016/j.jbc.2022.101646 (PMC8881472; doi:10.1016/j.jbc.2022.101646)
Supplement: Supplemental Figure S1 [file mmc1.docx]

**Supporting Information**

**Pharmacological chaperones for the oxytocin receptor increase oxytocin responsiveness in myometrial cells**

Running title: Pharmacological chaperones for oxytocin receptor

Manasi Malik^1^, Yingye Fang^2^, Monali Wakle-Prabagaran^1^, Michelle Roh^1^, Kevin Prifti^1^, Antonina I. Frolova^1^, Princess I. Imoukhuede^2^, Sarah K. England*^1^

^1^ Center for Reproductive Health Sciences, Department of Obstetrics and Gynecology, Washington University in St. Louis, St. Louis, Missouri, USA

^2^ Department of Biomedical Engineering, Washington University in St. Louis, St. Louis, Missouri, USA

^*^To whom correspondence should be addressed:

Sarah K. England. Department of Obstetrics and Gynecology, Washington University in St. Louis, School of Medicine, 425 South Euclid Avenue, Campus Box 8064, St. Louis, MO 63110, USA.

Email: englands@wustl.edu

https://orcid.org/0000-0003-4247-0281

**Table of Contents**

Figure S1: *Effects of V281M mutation and chaperone treatment on basal and maximal IP1 concentration* 2

Figure S1. *Effects of V281M mutation and chaperone treatment on basal and maximal IP1 concentration.* (A) IP1 concentration in unstimulated cells (no oxytocin treatment). (B) IP1 concentration in cells treated with 10 μM oxytocin. **P*<0.05, ***P<*0.005, *****P<*0.0001 by one-way ANOVA with Šidák’s multiple comparisons test; ns, not significant. Data shown are mean and standard error from *N*=5 independent trials.
